# Photodynamic and Cytotoxic Evaluation of *Euphorbia anacampseros* Methanolic Extract: Chemical Characterization, Molecular Docking, and Light‐Activated Anticancer Activity

**DOI:** 10.1002/cbdv.71897

**Published:** 2026-10-30

**Authors:** Sercin Ozlem Caliskan, Metin Caliskan, Emine İncilay Torunoğlu, Abidin Gümrükçüoğlu, Süleyman Doğu, Erdi Can Aytar

**Affiliations:** ^1^ Faculty of Medicine, Department of Biophysics Usak University Usak Türkiye; ^2^ Faculty of Medicine, Department of Medical Biology Usak University Usak Türkiye; ^3^ Faculty of Medicine, Department of Medical Biochemistry Necmettin Erbakan University Konya Türkiye; ^4^ Medicinal‐Aromatic Plants Application and Research Center Artvin Çoruh University Artvin Türkiye; ^5^ Meram Vocational School Konya Necmettin Erbakan University Konya Türkiye; ^6^ Medical and Cosmetic Plants Application and Research Center Konya Türkiye; ^7^ Faculty of Agriculture, Department of Horticulture Usak University Usak Türkiye

**Keywords:** epicatechin, *Euphorbia anacampseros*, molecular docking, photosensitizer, rutin

## Abstract

This study aimed to characterize the chemical constituents of the methanolic extract of *Euphorbia anacampseros* using high‐performance liquid chromatography (HPLC–DAD) and gas chromatography–mass spectrometry (GC–MS) analyses. The cytotoxic activity of the extract was assessed using the MTT assay, and its light‐enhanced cytotoxic effects were evaluated to investigate its potential for future photodynamic applications. Additionally, molecular docking analysis was performed to evaluate interactions between the identified compounds and the porphobilinogen deaminase (PBGD) enzyme. The mean inhibitory concentration (IC_50_) values were 54.72 µg/mL for HT‐29, 50.34 µg/mL for MCF‐7, and 58.97 µg/mL for A549 cell lines. In the dark, the extract exhibited moderate and nonselective baseline cytotoxicity across all tested cell lines, with IC_50_ values ranging from 46.95 to 58.97 µg/mL. However, the cytotoxic activity of the extract in HT‐29 cells was significantly enhanced following irradiation with blue, green, yellow, or red light, suggesting its potential for future photodynamic applications. Molecular docking targeting PBGD revealed that rutin and epicatechin exhibited the highest binding affinities, with binding energies of –9.9 and –7.8 kcal/mol, respectively. These findings suggest that *E. anacampseros* constituents may act as photosensitizing agents, potentially enhancing photodynamic therapy through interactions with enzymes involved in porphyrin metabolism.

## Graphical abstract

Methanolic extract of *Euphorbia anacampseros* is chemically characterized by HPLC–DAD and GC–MS analyses, and its photodynamic anticancer potential is evaluated against HT‐29, MCF‐7, and A549 cells under different light wavelengths. Molecular docking reveals favorable interactions between major phytochemicals and human porphobilinogen deaminase, supporting the extract as a promising source of bioactive compounds.

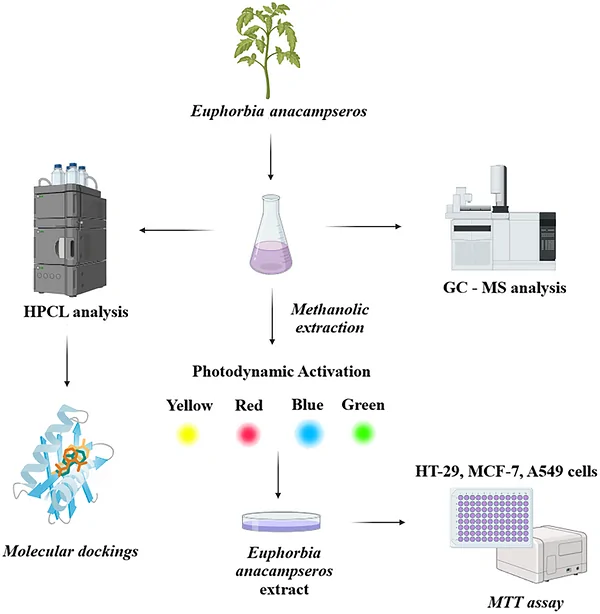

## 1 Introduction

Cancer is a heterogeneous group of diseases characterized by uncontrolled cell proliferation, invasion of surrounding tissues, and the ability to metastasize to distant organs [1]. Although there has been a noticeable improvement in cancer treatment outcomes in recent years, there is still a need for more effective, cost‐efficient, and less harmful therapeutic approaches. Unlike chemotherapy, which affects both tumor and healthy cells, there is a growing demand for therapies that act only at the tumor site, do not cause radiation‐related destructive effects like radiotherapy, and, most importantly, are not influenced by tumor heterogeneity. One such promising alternative is photodynamic therapy (PDT), whose significance has been increasingly recognized in recent years.

PDT is an alternative therapeutic strategy that mediates cell death mechanisms through the generation of reactive oxygen species (ROS), using a photosensitizer (PS) activated in the presence of light and molecular oxygen [2]. PDT requires the combination of three distinct components: a PS, light, and molecular oxygen. While these components are individually nontoxic, their combination leads to the formation of cytotoxic ROS [3]. PSs are chemical compounds capable of absorbing and storing light energy at specific wavelengths. The absorbed energy promotes these molecules to an exciting triplet state via intersystem crossing. Subsequently, the stored energy is transferred to molecular oxygen, leading to the generation of ROS [4].

The intracellular increase in ROS disrupts cellular homeostasis and triggers cell death mechanisms. The PS agent, which is nontoxic to normal cells at appropriate doses, is only activated in the presence of sufficient oxygen and upon exposure to suitable light, and its effect is restricted to the illuminated area. Unlike the systemic and severe chemical or physical effects caused by chemotherapy and radiotherapy, PSs disturb cellular homeostasis only at the application site, leading to programmed cell death or necrosis [5]. This makes them the key component of PDT. Thanks to the availability of advanced endoscopic techniques, PDT appears particularly advantageous for gastrointestinal cancers in terms of applicability, and it stands out as a safer, more affordable, and noninvasive treatment modality compared to conventional cancer therapies [6].

To date, more than 400 compounds have been identified as potential PSs for use in PDT [7]. Among the first‐generation PSs, hematoporphyrin derivatives (HPD; photofrin, porfimer sodium) have been used for the treatment of bladder cancer, early‐stage lung cancer, and esophageal cancer [8]. However, these PSs exhibit several important limitations, including limited tissue penetration, chemical heterogeneity, low light absorption capacity, and prolonged biological half‐life. Their long retention in tissues may lead to undesirable phototoxicity that can persist for several weeks after treatment. These limitations prompted the development of second‐generation PSs with improved photophysical properties [9].

Among the second‐generation PSs, 5‐aminolevulinic acid (5‐ALA, Levulan) has been used for the treatment of actinic keratosis [10], while *meta*‐tetra(hydroxyphenyl) chlorin (mTHPC; Foscan, temoporfin) has been used for the treatment of head and neck squamous cell carcinoma [11]. Second‐generation PSs provide improved tissue penetration through their enhanced light absorption ability and exhibit fewer side effects. However, they can still cause damage to healthy tissues because of their toxicity and display low chemical stability. Therefore, these limitations have led to the development of third‐generation PSs [9].

*N*‐aspartyl chlorin e6 (NPe6; talaporfin sodium, laserphyrin) is a representative third‐generation PS that has been used for the treatment of early‐stage lung cancer [12]. Third‐generation PSs are modified forms of second‐generation PSs. These modifications involve conjugation with components such as antibodies, amino acids, carbohydrates, or peptides. Another modification strategy is the encapsulation of second‐generation PSs into carrier systems, including liposomes, nanoparticles, and micelles [9].

Natural products have attracted considerable attention for use in PDT because they exhibit fewer or no side effects compared with synthetic drugs and support environmentally friendly, green approaches. Examples of these natural products include curcumin, anthraquinones, tolyporphin, hypericin, thiophenes, and hypocrellin. Medicinal plants containing bioactive compounds are regarded as natural sources of PSs. Previous studies have reported that these medicinal plants possess photoactive properties and are considered promising third‐generation PSs [7].

The Euphorbiaceae are predominantly distributed in tropical and subtropical regions of the world and comprise 6745 taxa belonging to 317 genera [13]. Among the species of the genus *Euphorbia*, only approximately 250 have been chemically and pharmacologically investigated [14, 15].

Members of this genus have been traditionally used in China, Europe, America, Turkey, India, Africa, Iran, and Pakistan [15]. The stems, leaves, roots, and milky latex of *Euphorbia* are widely employed in the treatment of respiratory infections, skin and body irritations, digestive disorders, inflammatory conditions, general body pain, and cancer. Some diterpenoids isolated from Euphorbia species have been reported to exhibit cytotoxic activity against various cancer types [16, 17].

Approximately 90 *Euphorbia* taxa are naturally distributed in Turkey, more than 40% of which are endemic. Their distribution across the country is mainly concentrated in three regions: the Mediterranean, Central Anatolia, and Eastern Anatolia. The most common species include *Euphorbia rigida* Bieb., *Euphorbia macroclada* Boiss., *Euphorbia myrsinites* L., *Euphorbia seguieriana* Neck., and *Euphorbia chamaesyce* L. The distribution of these species exhibits a wide geographical range, including steppe regions, forest valleys, coastal, and stony areas [18].

*Euphorbia anacampseros* Boiss. var. *anacampseros*, is distributed in diverse habitats such as steppes, forest valleys, coastal areas, and rocky terrains. *E. anacampseros* is a member of the genus *Euphorbia* within the Euphorbiaceae, which belongs to the order Malpighiales. *E. anacampseros* is a succulent species that typically grows as a ground cover or develops in the form of a small shrub [19].

Species belonging to the *Euphorbia* genus have been widely used in cancer treatment. To the best of our literature search, no previous study has investigated *E. anacampseros*. Therefore, this study aimed to evaluate the cytotoxic effect and the potential of *E. anacampseros* for use in PDT. The cytotoxic and photodynamic effects of *E. anacampseros* extract were evaluated against MCF‐7 breast cancer, HT‐29 colon cancer, and A549 lung cancer cell lines. Finally, in silico studies were performed to elucidate its possible molecular targets.

## 2 Materials and Methods

### 2.1 Plant Collection

The specimens of *Euphorbia anacampseros* Boiss. *var. anacampseros*, selected for the study, were collected from C4 KONYA: Konya–Beyşehir Road, Değirmenköy road junction, roadside, 1300 m, 02.06.2025, S. DOGU 3150. The plant material was identified by Dr. Süleyman Doğu. The voucher specimens were dried according to standard herbarium procedures and deposited as herbarium specimens. These specimens were deposited in the Necmettin Erbakan University Ahmet Keleşoğlu Faculty of Education Herbarium. A portion of the collected material was stored under dark and dry conditions of light‐free and moisture‐free conditions, and stored at 4°C, for utilization in the present study.

### 2.2 Plant Extraction

*E. anacampseros* were harvested and thoroughly washed with distilled water. After cleaning, the plant material was dried at 40°C for 48 h and ground into a fine powder using a blender. The powdered sample was mixed with methanol at a 1:10 (w/v) ratio and subjected to maceration for 72 h. Following extraction, the mixture was filtered, and the methanolic phase was concentrated under reduced pressure at 40°C using a rotary evaporator. Extraction of 30 g of dried powdered plant material with methanol, followed by solvent removal, yielded 2.32 g of crude extract, corresponding to an extraction yield of 7.73% (w/w). Owing to the limited amount of available plant material, the extraction was performed as a single batch. A portion of the obtained crude extract was dissolved in methanol to prepare a stock solution for subsequent analyses. The crude extract was stored in airtight containers in the dark at 4°C to minimize the degradation of light‐sensitive constituents and was analyzed immediately after extraction [20].

### 2.3 Reference Standards and Calibration

Phenolic compounds, flavonoids, and vitamin C reference standards were analyzed under two different analytical conditions. Quantitative determination was performed using six‐point external calibration curves (25–300 µg/mL), and all calibration standards were analyzed by high‐performance liquid chromatography with diode array detection (HPLC–DAD) under the same instrumental conditions as the samples.

Under Study A, the following standards were used: L‐ascorbic acid, gallic acid, 3,4‐dihydroxybenzoic acid, (+)‐catechin, (−)‐epicatechin, trans‐caffeic acid, vanillic acid, rutin, p‐coumaric acid, ferulic acid, rosmarinic acid, myricetin, quercetin, and apigenin. Under Study B, the standards included pyrogallol, chlorogenic acid, syringic acid, cyanidin chloride, resveratrol, oleuropein, hesperetin, kaempferol, baicalein, and chrysin.

Compound identity was confirmed by matching both the retention times (RT) and the DAD UV spectra of sample peaks with those of the corresponding reference standards. All calibration curves were linear over the studied range, with correlation coefficients (*R* ^2^) ranging from 0.9940 to 0.9999.

### 2.4 High‐Performance Liquid Chromatography (HPLC)‐Based Compound Characterization

To profile the selected compounds, two separate HPLC methods were employed. The first method (Study A) was used to analyze the initial 14 compounds, while the second (Study B) was dedicated to the remaining 10. Both analyses were performed using an ACE 5 C18 reversed‐phase column (250 mm × 4.6 mm, 5 µm particle size).

For Study A, the mobile phase comprised acetonitrile (Solvent A) and 1.5% aqueous acetic acid (Solvent B), with a gradient program initiating at 15% A and 85% B and progressing to 40% A and 60% B by the 29th min. In Study B, methanol replaced acetonitrile as Solvent A, and the gradient started at 10% A and 90% B. It reached 40% A by 29 min, increased to 60% A at 40 min, and finally to 90% A by 53 min.

Both chromatographic methods were executed on an Agilent 1260 Infinity II HPLC system, comprising a quaternary pump (flow rate: 0.7 mL/min), a G7116A column oven set to 35°C, and a vial sampler with a 10 µL injection volume. Detection was performed using a diode array detector (1260 DAD WR), set to monitor absorbance at 250, 270, and 320 nm for Study A, and at 280, 290, 320, 370, and 535 nm for Study B.

Each compound was quantified using the monitored wavelength corresponding to its absorption maximum. Sample solutions were diluted prior to injection so that the response of each analyte fell within the calibrated range, and the measured concentrations were backcalculated using the corresponding dilution factors. Representative chromatograms are presented in Figure S1.

**TABLE 1:** Quantitative analysis of phenolic and flavonoid compounds in *E. anacampseros* extract.

Nos.	Compounds	*E. anacampseros* (mg/L)
**Vitamin**		
**1**	L‐Ascorbic acid	7460.1
**Phenolics**		
**2**	Gallic acid	320.3
**3**	3,4‐Dihydroxy benzoic acid	N/D
**4**	Vanillic acid	780.1
**5**	Syringic acid	N/D
**6**	p‐Coumaric Acid	108.3
**7**	trans‐Caffeic acid	106.3
**8**	Ferulic acid	1.8
**9**	Rosmarinic acid	34.9
**10**	Pyrogallol	1387.3
**11**	Chlorogenic acid	201.2
**12**	Resveratrol	N/D
**13**	Oleuropein	N/D
**Flavonoids**		
**14**	(+)‐Catechin	1265.9
**15**	(‐)‐Epicatechin	2296.1
**16**	Rutin	4664.5
**17**	Myricetin	N/D
**18**	Quercetin	76.2
**19**	Apigenin	N/D
**20**	Cyanidin chloride	N/D
**21**	Hesperetin	N/D
**22**	Kaempferol	N/D
**23**	Baicalein	34.2
**24**	Chrysin	N/D

### 2.5 Gas Chromatography–Mass Spectrometry (GC–MS) Analysis of Volatile Compounds

Volatile compound analysis was performed using gas chromatography–mass spectrometry (GC–MS) following solid‐phase microextraction (SPME). Plant samples were homogenized with a laboratory‐grade grinder and loaded into 20 mL headspace vials (Supelco) fitted with PTFE/silicone septa, to approximately one‐third of the vial volume. The vials were incubated at 45°C for 15 min to enhance volatile emission.

After incubation, a preconditioned SPME fiber was inserted into the vial headspace for 40 min to adsorb volatiles. The fiber was then transferred to the GC inlet for thermal desorption over a 20‐min period.

GC–MS analysis was conducted using an Agilent GC system (Agilent Technologies, Santa Clara, CA, USA) with an HP‐5MS ultra inert capillary column (30 m × 0.25 mm × 0.25 µm). High‐purity helium (≥ 99.99%) served as the carrier gas at a constant flow of 1.0 mL/min. The injector temperature was maintained at 250°C. The oven temperature program began at 50°C (held for 2 min), followed by a temperature ramp of 2.5°C/min up to 150°C (held for 5 min), and then increased at 6.5°C/min to 250°C, followed by a 1‐min final hold. The MS detector operated in electron impact (EI) mode at 70 eV, scanning over an *m*/*z* range of 35–500. Retention index (RI) values of the compounds were calculated using a series of *n*‐alkanes (C7–C30) analyzed under the same conditions. Compound identification was based on the comparison of mass spectra with the NIST library (similarity scores ranging from 80% to 97%) together with the comparison of the experimental RI values with the library RI values (RI*).

A representative total ion chromatogram is provided in Figure S2.

### 2.6 Preparation of *E. anacampseros* Extract Dilutions

*E. anacampseros* extract was dissolved in methanol to prepare a 100 mg/mL stock solution. To mitigate the cytotoxic effects of methanol, this stock solution was further diluted with culture medium to achieve a final concentration of 1536 µg/mL. Serial dilutions were then performed from this working solution to obtain final concentrations of 768, 384, 192, 96, 48, 24, and 12 µg/mL. The stock solution and subsequent dilutions were used to assess their effects on cultured cells.

### 2.7 Assessment of the Anticancer Activity of the *E. anacampseros* Extract

Human embryonic kidney cell line HEK293 (RRID: CVCL_0045), human colorectal adenocarcinoma cell line HT‐29 (RRID: CVCL_0320), human breast adenocarcinoma cell line MCF‐7 (RRID: CVCL_0031), and human lung carcinoma cell line A549 (RRID: CVCL_0023) were used in this study. The cell lines were originally obtained from the American Type Culture Collection (ATCC) and were maintained in the cell culture archive of the Department of Basic Medical Sciences, Faculty of Medicine, Uşak University, Turkey. HEK293 and MCF‐7 cells were cultured in Dulbecco's Modified Eagle Medium (DMEM, high glucose) (Gibco, USA), while A549 and HT‐29 cells were cultured in RPMI‐1640 medium (Gibco, USA). Both media were supplemented with 10% fetal bovine serum (FBS) (Gibco, USA) and 1% penicillin/streptomycin (Gibco, USA). The culture process was carried out in a humidified incubator at 37°C with 5% CO_2_. When the cells reached 85%–90% confluence, cells were detached using trypsin‐EDTA (Sigma–Aldrich) and subsequently seeded into 96‐well plates at a density of 1 × 10^4^ cells per well. Separate plates were prepared for anticancer activity and photodynamic activity assessments. The plates were then incubated for 24 h. After incubation, the supernatants from all wells were discarded, and only culture medium (200 µL) was added to the control wells, while extract dilutions (200 µL) were added to the anticancer activity plates in triplicate. The plates were then incubated for 72 h.

### 2.8 Assessment of the Photodynamic Activity of the *E. anacampseros* Extract

As in the anticancer activity assay, after a 24‐h incubation, the supernatants from the photodynamic activity assessment plates were completely discarded. Only culture medium (200 µL) was added to the control wells, while extract dilutions (200 µL) were added in triplicate. Methanol vehicle controls were included for all tested concentrations to exclude potential solvent‐induced toxicity. No significant reduction in cell viability was observed in vehicle control groups under dark or light conditions. Following a 50‐min incubation, the supernatant was removed from all wells, and fresh medium (50 µL) was added to each well. A three‐panel LED system (O'melon Omega LED), consisting of 287 bulbs and capable of emitting light at four different wavelengths of blue (423 nm), yellow (583 nm), red (640 nm), and green (532 nm), was used as the light source. The LED system is a low‐power irradiation device that provides nonthermal visible light exposure. Spectrophotometric analysis revealed that the *E. anacampseros* extract did not exhibit a distinct absorbance peak within the visible spectrum. Therefore, all four wavelengths provided by the system were tested. Light output was measured using a power meter (Newport, USA), and over the 50‐min exposure period, irradiance and fluence values were obtained as follows: red light –0.610 mW/cm^2^ irradiance and 1.83 J/cm^2^ fluence; yellow light –0.674 mW/cm^2^ irradiance and 2.02 J/cm^2^ fluence; blue light –0.836 mW/cm^2^ irradiance and 2.5 J/cm^2^ fluence; green light –0.479 mW/cm^2^ irradiance and 1.43 J/cm^2^ fluence. Due to the very low irradiance of the LED system (0.479–0.836 mW/cm^2^), no apparent heating of the samples was observed during irradiation. For each wavelength, separate plates containing extract‐treated wells and light‐only control wells were exposed to the corresponding LED light for 50 min. Another plate was kept in complete darkness under the same conditions. At the end of the exposure period, the supernatant was discarded from all wells, and each well was replenished with 200 µL of fresh culture medium. The plates were then incubated for 48 h. Each experiment was performed using three biological replicates, each with three technical replicates.

### 2.9 MTT Assay for Determining Cell Viability

Following the completion of the incubation periods for all experimental groups, the supernatant was removed from all wells, and 50 µL of MTT solution was added to each well together with 45 µL of culture medium. The plates were then incubated at 37°C for 4 h. At the end of the incubation period, 100 µL of 99.9% DMSO was added to dissolve the formazan crystals, and the plates were incubated for an additional hour. Cell viability was assessed at 570 nm using a Multiskan Sky Microplate Spectrophotometer (Thermo Fisher Scientific). The percentage of cell viability was determined based on optical density (OD) values using the following formula.

$$\mathrm{Cell}\;\mathrm{viability}\left(\%\right)=\left(OD_{\mathrm{sample}}/OD_{\mathrm{control}}\right)\times 100$$

### 2.10 Molecular Docking Analysis

Molecular docking analysis was performed to investigate the molecular interactions and binding affinities of the major compounds identified by HPLC–DAD and GC–MS analyses toward human porphobilinogen deaminase (PBGD). The docking study was conducted to evaluate the potential binding modes of the selected ligands within the active site of the target enzyme and to characterize the molecular interactions responsible for their predicted binding affinities.

The 3D crystal structure of human porphobilinogen deaminase (PDB ID: 3ECR) was retrieved from the Protein Data Bank (PDB). Protein preparation was performed by removing crystallographic water molecules and nonessential heteroatoms, followed by the addition of polar hydrogen atoms and assignment of Gasteiger partial charges using AutoDock Tools. The prepared protein structure was subsequently converted into PDBQT format.

The ligands identified by HPLC–DAD and GC–MS analyses were retrieved from the PubChem database in SDF format and converted into PDB format using BIOVIA Discovery Studio Visualizer 2021. The ligand structures were subsequently prepared in AutoDock Tools by adding polar hydrogen atoms, assigning Gasteiger partial charges, defining rotatable bonds, and converting them into PDBQT format.

The docking grid was centered on the putative active site of human PBGD at *x* = 9, *y* = 31, and *z* = 36 Å. Molecular docking simulations were carried out using AutoDock Vina version 1.2.5. For each ligand, nine binding poses (num_modes = 9) were generated, and the conformation exhibiting the lowest predicted binding free energy was selected for subsequent interaction analysis.

To validate the docking protocol, the co‐crystallized ligand was redocked into the binding site of human PBGD (PDB ID: 3ECR). The calculated root‐mean‐square deviation (RMSD) value was 1.05 Å, confirming the reliability of the docking protocol for reproducing the experimental ligand‐binding conformation.

The 3D binding conformations and 2D protein–ligand interaction diagrams were analyzed using BIOVIA Discovery Studio Visualizer 2021. Binding affinities, hydrogen bonding, hydrophobic interactions, π‐interactions, and the amino acid residues involved in ligand binding were comprehensively evaluated to characterize the predicted interactions between the selected ligands and human PBGD.

### 2.11 Determination of IC_50_ Values and Statistical Analysis

The IC_50_ value, representing the extract concentration required to inhibit cell viability by 50%, was determined using GraphPad Prism software. IC_50_ values are reported along with their corresponding 95% confidence intervals (CI). The statistical significance of viability percentages at the same concentrations was assessed using one‐way ANOVA in GraphPad Prism software, followed by Tukey's post hoc test. A significance threshold of *p* < 0.05 was applied, and *p*‐values below this threshold were considered statistically significant. To evaluate the preferential toxicity of the extract toward cancer cells compared to normal cells, the Selectivity Index (SI) was calculated using the following formula: SI = IC_50_ of normal cells (HEK293)/IC_50_ of cancer cells (HT‐29). An SI value greater than 1.0 indicates a higher degree of selectivity toward cancer cells.

## 3 Results and Discussion

### 3.1 HPLC‐Based Phytochemical Profiling of *E. anacampseros* Extract

The HPLC–DAD chromatograms of the extract are presented in Figure S1; the annotated peaks correspond to the compounds listed in Table 1.

HPLC analysis revealed the presence of various bioactive compounds in the extract of *E. anacampseros*, including vitamin C, phenolic acids, and flavonoids. L‐Ascorbic acid was detected at the highest concentration (7460.1 mg/L), suggesting a strong antioxidant capacity. Among the phenolic acids, pyrogallol (1387.3 mg/L), vanillic acid (780.1 mg/L), and gallic acid (320.3 mg/L) were the most abundant, followed by chlorogenic acid (201.2 mg/L), p‐coumaric acid (108.3 mg/L), trans‐caffeic acid (106.3 mg/L), rosmarinic acid (34.9 mg/L), and ferulic acid (1.8 mg/L). Several phenolic compounds, including 3,4‐dihydroxybenzoic acid, syringic acid, resveratrol, and oleuropein, were not detected in the extract.

In terms of flavonoids, rutin (4664.5 mg/L), (–)‐epicatechin (2296.1 mg/L), and (+)‐catechin (1265.9 mg/L) were identified at relatively high concentrations, together with quercetin (76.2 mg/L) and baicalein (34.2 mg/L). Conversely, other flavonoids such as myricetin, apigenin, hesperetin, kaempferol, and chrysin were not detected under the chromatographic conditions applied. These findings indicate that *E. anacampseros* possesses a phytochemical profile rich in phenolic compounds that may contribute to its biological activity. which may contribute to its potential biological activities.

### 3.2 Volatile Compound Profiling of *E. anacampseros* Extract via GC–MS Analysis

The total ion chromatogram is presented in Figure S2; the annotated peaks correspond to the compounds listed in Table 2.

**TABLE 2:** Volatile compound profiling of *E. anacampseros* extract via GC–MS analysis.

Nos.	RT (min)	RI	RI*	Match (%)	Name of the compound	Content (%)
**1**	6.046	797	800	80	Hexanal	25.527
**2**	7.635	846	855	97	2‐Hexenal, (E)‐	17.652
**3**	10.663	925	930	85	2,4,6‐Octatrienal	17.257
**4**	11.455	949	944	90	Isocumene	3.055
**5**	11.735	956	961	93	Benzaldehyde	12.426
**6**	12.451	973	978	90	beta‐Pinene	5.951
**7**	12.899	983	988	94	Sulcatone	9.750
**8**	14.564	1019	1026	95	p‐Cymene	8.382

Rt: retention times on an HP‐5MS UI column.

RI: experimentally determined retention indices on an HP‐5MS UI column.

RI*: reference retention index values obtained from the NIST library.

Match (%): mass‐spectral similarity to the NIST library reference spectrum.

Content (%): expressed as a percentage of the total identified peak area.

GC–MS analysis of the *E. anacampseros* extract resulted in the identification of eight volatile compounds. The most abundant compound was hexanal, detected at 25.527%. Other identified compounds included (E)‐2‐hexenal (17.652%), 2,4,6‐octatrienal (17.257%), benzaldehyde (12.426%), sulcatone (9.750%), p‐cymene (8.382%), β‐pinene (5.951%), and isocumene (3.055%). The RT of the compounds ranged from 6.046 to 14.564 min, while their respective RI values varied between 797 and 1019. Compound identification was based on the comparison of RT and RI data.

### 3.3 Anticancer Activity and Photodynamic Therapy

Under dark conditions, the methanolic extract of *E. anacampseros* demonstrated moderate baseline cytotoxicity. The IC_50_ values were 46.95 µg/mL for HEK293, 54.72 µg/mL for HT‐29, 50.34 µg/mL for MCF‐7, and 58.97 µg/mL for A549 (Figure 1). Statistical analysis (one‐way ANOVA) revealed that the differences between these baseline IC_50_ values were not statistically significant (*p* > 0.05), indicating that the extract lacks inherent biological selectivity in the absence of light activation.

**FIGURE 1:**
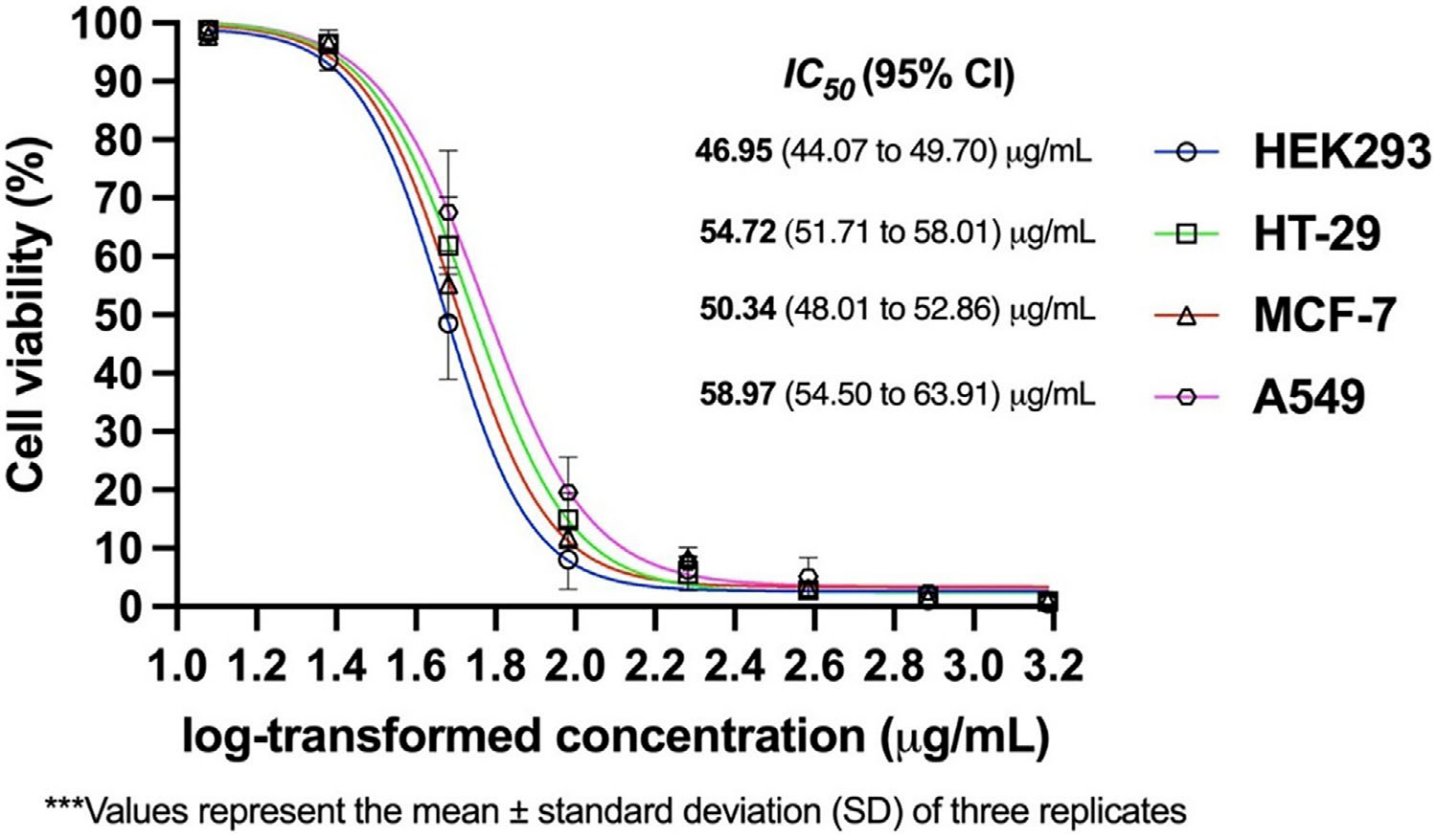
Relative cell viability (%) of HEK293, HT‐29, MCF‐7, and A549 cells in response to increasing concentrations of the *E. anacampseros* extract under dark conditions. Data points represent the mean ± SD of three independent biological replicates, with each performed in triplicate (technical replicates). Error bars indicate the SD. Values are presented as a function of log‐transformed concentrations and mean IC_50_ values are reported with their corresponding 95% CI.

The photodynamic efficacy of *E. anacampseros* extract significantly increased in both HEK293 (normal) and HT‐29 (colorectal cancer) cell lines when combined with light exposure at different wavelengths (blue, green, yellow, and red) (Table S1).

Compared to the control groups, IC_50_ values were significantly reduced in all light‐treated groups, indicating that the cytotoxic effect of the extract was enhanced through light activation. In HEK293 cells, treatment with red light resulted in the lowest IC_50_ value (110.6 µg/mL), indicating the highest cytotoxic effect. Green, yellow, and blue light treatments also exhibited cytotoxic activity with IC_50_ values of 128.8, 149.2, and 161.7 µg/mL, respectively.

Similarly, in HT‐29 cells, the combination with yellow light produced the most pronounced cytotoxic effect, yielding an IC_50_ value of 194.3 µg/mL. Green, red, and blue light treatments also demonstrated cytotoxicity, with IC_50_ values of 222.2, 253.6, and 281.8 µg/mL, respectively (Figure 2).

**FIGURE 2:**
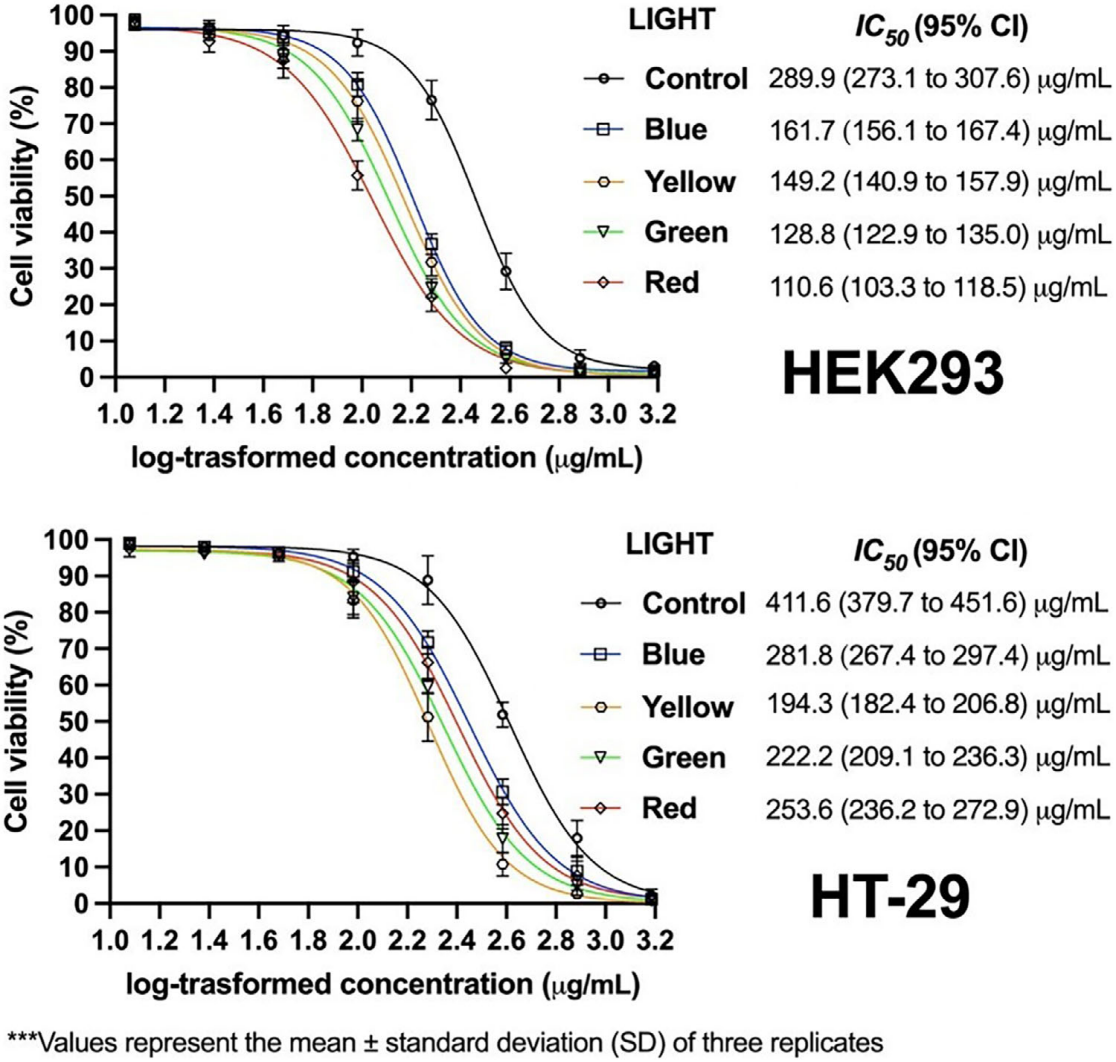
Relative cell viability (%) of HEK293 and HT‐29 cells following exposure to blue (423 nm), green (532 nm), yellow (583 nm), and red (640 nm) light in the presence of *E. anacampseros* extract. Data points represent the mean ± SD derived from three independent biological replicates, each conducted in triplicate (technical replicates). Error bars represent the SD. Results are presented as a function of log‐transformed concentrations, and IC_50_ values are shown along with their 95% CI.

Based on the calculated IC_50_ values, the SI was determined to be 0.86 under dark conditions (46.95 µg/mL for HEK293/54.72 µg/mL for HT‐29). Under yellow light irradiation, which yielded the highest cytotoxic effect in HT‐29 cells, the SI was 0.77 (149.2 µg/mL for HEK293/194.3 µg/mL for HT‐29). These values indicate that the crude methanolic extract currently lacks inherent biological selectivity toward the tested adenocarcinoma cell line in vitro.

To evaluate the anticancer activity, cancer cell lines with the highest global incidence and mortality rates, namely A549, MCF‐7, and HT‐29, were selected. For assessing photodynamic activity, the HT‐29 cell line was specifically chosen owing to the availability of advanced endoscopic techniques.

Although the potential of plant extracts as sources of PSs in PDT has rarely been investigated, their chemotherapeutic properties have been studied [7]. The identification of new potential PSs represents a critical first step in PDT research [21]. Currently, only a limited number of PDT drugs are available, including photofrin, foscan, and levulan, which are mainly used in the treatment of skin, gynecological, gastrointestinal, and head and neck cancers [8].

In this study, an extract from *E. anacampseros*, which did not exhibit toxicity against HT‐29, MCF7, and A549 cancer cell lines under normal conditions, was investigated. Therefore, the aim of this study was to evaluate the applicability of *E. anacampseros* extracts in PDT. Notably, the extract exhibited light‐induced cytotoxicity at various wavelengths (blue, green, yellow, and red). The IC_50_ values were significantly reduced in all light‐treated groups compared to the control groups, indicating that the cytotoxic effect of the extract was enhanced by light activation. Although statistically significant differences were observed among the applied wavelengths, these differences were not considered to be of substantial biological relevance. The present findings suggest that one or more compounds within the *E. anacampseros* extract possess photosensitizing properties. Further in vivo studies are warranted to confirm these findings.

Previous studies have demonstrated the anticancer activity of *Euphorbi*a species extracts [22]. However, to date, no anticancer studies have been conducted specifically on *E. anacampseros*. This study aims to investigate the anticancer properties of *E. anacampseros* on HT‐29, MCF7, and A549 cancer cell lines, as well as its potential for future photodynamic applications.

Although numerical differences in sensitivity were observed in the dark (particularly a slightly lower IC_50_ value for HEK293 compared to cancer cell lines), these differences were not statistically significant. Such differences likely reflect stronger survival and resistance mechanisms and different metabolic profiles in adenocarcinoma cells compared to the normal HEK293 cell line. Consequently, the crude extract does not exhibit selective anticancer activity under normal conditions; however, the observation of a significant, wavelength‐dependent decrease in IC_50_ values upon light irradiation supports its potential as a photodynamic candidate. A PS is an agent that becomes cytotoxic upon activation by light of a specific wavelength [23]. Among the treated groups, red‐light treatment of HEK293 cells produced the lowest IC_50_ value (110.6 µg/mL), indicating the highest cytotoxic effect. Similarly, in HT‐29 cells, the combination with yellow light was the most effective, with an IC_50_ value of 194.3 µg/mL. Upon light exposure, the cytotoxic activity of the extract increased significantly. These findings indicate that the extract contains compounds capable of enhancing light‐dependent cytotoxicity and support further investigation of their potential application in PDT.

Recent studies have shown increasing interest in plant‐derived compounds and natural polyphenols as potential candidates for photodynamic applications. Natural products such as hypericin, curcumin, chlorophyll derivatives, quinonoids, and various plant extracts have been investigated because of their light‐responsive properties and biological compatibility; however, their photophysical characteristics and underlying mechanisms require compound‐specific validation [24, 25]. Flavonoids are of particular interest because they can exhibit both pro‐oxidant and antioxidant activities depending on their concentration, cellular environment, and irradiation conditions, thereby influencing ROS‐mediated cytotoxic responses [26]. In a study by Vaziri et al. [27], a rutin–gallium complex used in antimicrobial PDT induced a marked increase in intracellular ROS levels and significantly reduced the viability of *Streptococcus mutans* biofilms, demonstrating that chemical modification of rutin may confer measurable photosensitizing activity. Catechin has also been reported to enhance zinc phthalocyanine‐mediated PDT in A549 lung cancer cells by increasing cytotoxicity, DNA damage, and apoptosis compared with treatment using the PS alone [28]. Similarly, Chota et al. [29] demonstrated that the *Dicoma anomala* extract enhanced phthalocyanine‐mediated photodynamic effects in MCF‐7 breast cancer cells, suggesting that plant‐derived phytochemical constituents may contribute to light‐dependent anticancer activity. In the present study, rutin, epicatechin, and other phytochemical constituents identified in *E. anacampseros* may have contributed to the observed wavelength‐dependent light‐enhanced cytotoxicity. However, because isolated compounds and ROS‐related mechanisms were not experimentally evaluated, their individual contributions and potential photosensitizing roles remain to be elucidated in future studies.

Although the IC_50_ values reported for *Lumnitzera racemosa* (11.63 µg/mL) and *Albizia procera* (10.73 µg/mL) were substantially lower than those observed for *E. anacampseros* in the present study, direct quantitative comparison should be interpreted with caution because different plant species, extraction methods, cell models, irradiation parameters, and experimental protocols were used [30]. Nevertheless, both studies consistently demonstrate that plant‐derived extracts can exhibit enhanced cytotoxicity following visible light irradiation, supporting the potential of natural products as candidates for future photodynamic research.

Liao and colleagues reported that the combination of light with plant extracts from *Rhizoma coptidis* and *Cortex phellodendri* reduced cell viability and proliferation [31]. Similarly, our findings demonstrated that the combination of *E. anacampseros* extract with yellow light significantly reduced cell viability and proliferation in HT‐29 cells.

The extract of *E. anacampseros* contained various phenolic and flavonoid compounds such as L‐ascorbic acid (vitamin C), vanillic acid, pyrogallol, (+)‐catechin, (−)‐epicatechin, and rutin, as well as volatile compounds including hexanal, 2,4,6‐octatrienal, and benzaldehyde. These constituents may contribute to the photosensitizing activity of *E. anacampseros* [25]. The identification and isolation of the compounds responsible for photodynamic activity could enhance the photodynamic efficacy of the extract and support its potential as a novel natural source of PSs.

In the study conducted by Erdogan et al., silver nanoparticles (AgNPs) synthesized from *Cynara scolymus* leaf extracts exhibited broad‐spectrum anticancer activity against MCF‐7 breast cancer cells when applied in combination with PDT [32]. Similarly, in our study, the extract of *E. anacampseros* demonstrated anticancer activity when combined with light irradiation, consistent with these findings.

A study reported by Liu et al. demonstrated that berberine‐mediated PDT exerted antineoplastic effects on HeLa cells [33]. Likewise, Oliveira et al. reported the efficacy of berberine‐based PDT in the treatment of cervical carcinoma [34]. The effects of *E. anacampseros* extract observed in HT‐29 cells in our study are consistent with these findings in the literature, suggesting that it may be effective in anticancer therapy.

Koon et al. demonstrated that the combination of curcumin extract with light could enhance photosensitization in NPC/CNE2 cells [35]. In our study, *E. anacampseros* extract significantly reduced cell viability across all light groups (blue, green, yellow, and red), indicating that its cytotoxic effect was enhanced through light activation. Among these, the combination with yellow light yielded the most pronounced effect in HT‐29 cells.

Furthermore, Dujic et al. [36] reported that the photodynamic effect of curcumin significantly suppressed tumor growth in an A431 human epithelial carcinoma tumor model, whereas no reduction in tumor volume was observed in the control group. The use of curcumin in PDT has also been reported to exhibit its anticancer properties in breast cancer cells [37]. Unlike curcumin, which is a well‐characterized natural PS with defined photophysical properties, *E. anacampseros* is a complex phytochemical mixture. Therefore, the observed light‐enhanced cytotoxicity may result from the combined activity of multiple constituents rather than a single photoactive compound. Within the scope of our study, *E. anacampseros* extract reduced cell viability in HT‐29 cells following visible light exposure, while red light produced the lowest cytotoxicity in HEK‐293 cells. Although these findings support the light‐enhanced cytotoxic potential of the extract, further studies are required to identify the responsible photoactive constituents and clarify their mechanisms of action.

The comparable phototoxicity observed in HEK293 and HT‐29 cells, resulting in a narrow therapeutic window, represents a significant consideration for the potential clinical application of the crude extract. This lack of inherent biological selectivity poses a potential risk for systemic administration; however, the clinical relevance of PDT relies not only on biological selectivity but also on spatial selectivity, where the cytotoxic effect is strictly confined to the area of light irradiation. In clinical practice, particularly for gastrointestinal cancers like the HT‐29 model used here, advanced endoscopic techniques can be utilized to deliver light precisely to the tumor site, thereby minimizing damage to surrounding healthy tissues despite the observed low SI. To overcome current clinical limitations, future research should prioritize the isolation of specific bioactive compounds, such as rutin and epicatechin, which exhibited high binding affinity in our docking models, or the development of targeted delivery systems to improve cancer‐specific uptake and broaden the therapeutic window.

### 3.4 Binding Affinity and Inhibitory Potential of *E. anacampseros* Compounds via Molecular Docking

Molecular docking simulations were performed to evaluate the interaction potential of selected compounds identified in *E. anacampseros* extract with human porphobilinogen deaminase (PBGD; PDB ID: 3ECR) (Table 3). Among the tested molecules, rutin exhibited the strongest binding affinity with a binding energy of –9.9 kcal/mol (Figure 3), followed by epicatechin (–7.8 kcal/mol) (Figure 4) and ascorbic acid (–5.3 kcal/mol). In contrast, the aldehydic compounds hexanal (–3.8 kcal/mol), (E)‐2‐hexenal (–4.1 kcal/mol), and 2,4,6‐octatrienal (–4.8 kcal/mol) displayed comparatively weaker binding affinities. Ligand efficiency (LE) values ranged from 0.230 (rutin) to 0.585 ((E)‐2‐hexenal), whereas fit quality (FQ) values varied between 0.199 and 0.868. Although rutin exhibited the lowest LE value, it showed the highest FQ value together with the most favorable binding energy, indicating that it achieved the best overall balance between binding affinity and molecular size. Overall, rutin and epicatechin emerged as the most promising ligands against human PBGD based on their favorable binding affinities, while the remaining compounds exhibited relatively weaker interactions.

**TABLE 3:** Molecular docking results of selected compounds against human PBGD (3ECR).

	Binding energy (kcal/mol)	LE	FQ
**Hexanal**	−3.8	0.542	0.199
**2‐Hexenal, (E)‐**	−4.1	0.585	0.214
**2,4,6‐Octatrienal**	−4.8	0.533	0.309
**Ascorbic acid**	−5.3	0.441	0.406
**Rutin**	−9.9	0.230	0.868
**Epicatechin**	−7.8	0.371	0.704

Abbreviations: FQ, fit quality; LE, ligand efficiency.

**FIGURE 3:**
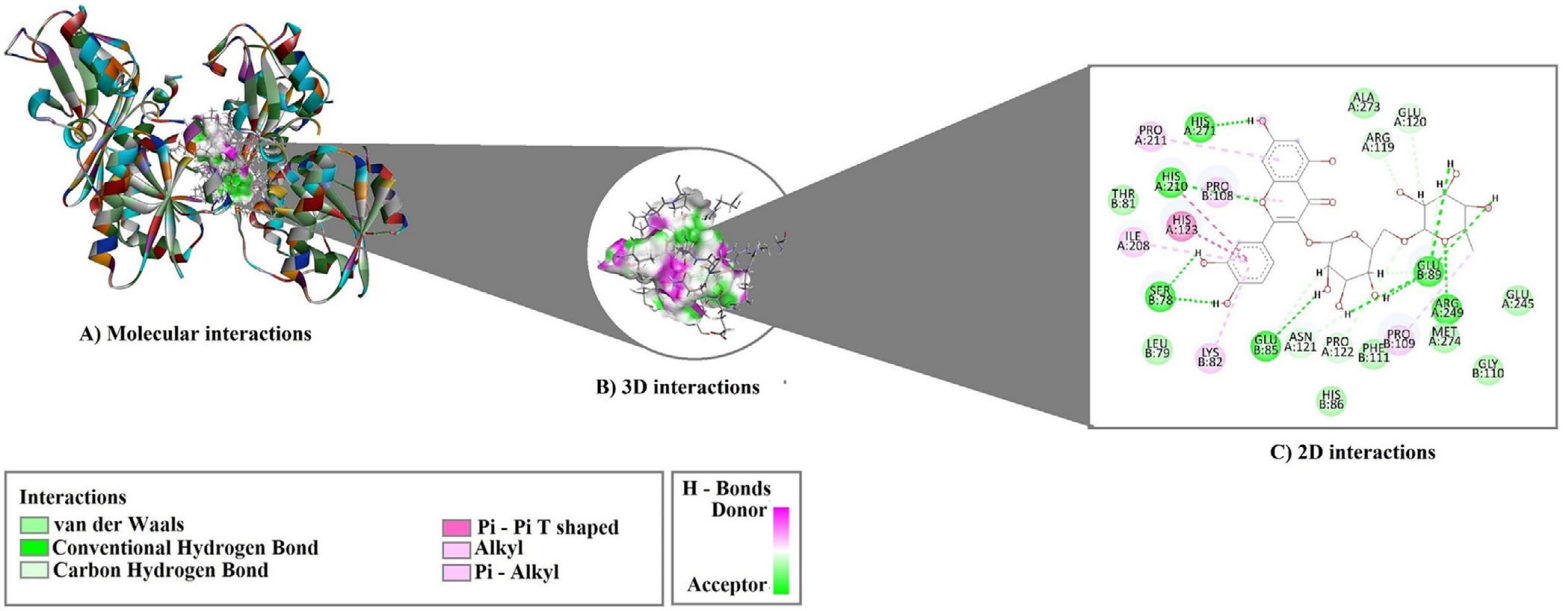
Molecular docking interactions of rutin with PBGD (3ECR): (A) molecular interactions, (B) 3D interactions, and (C) 2D interactions.

**FIGURE 4:**
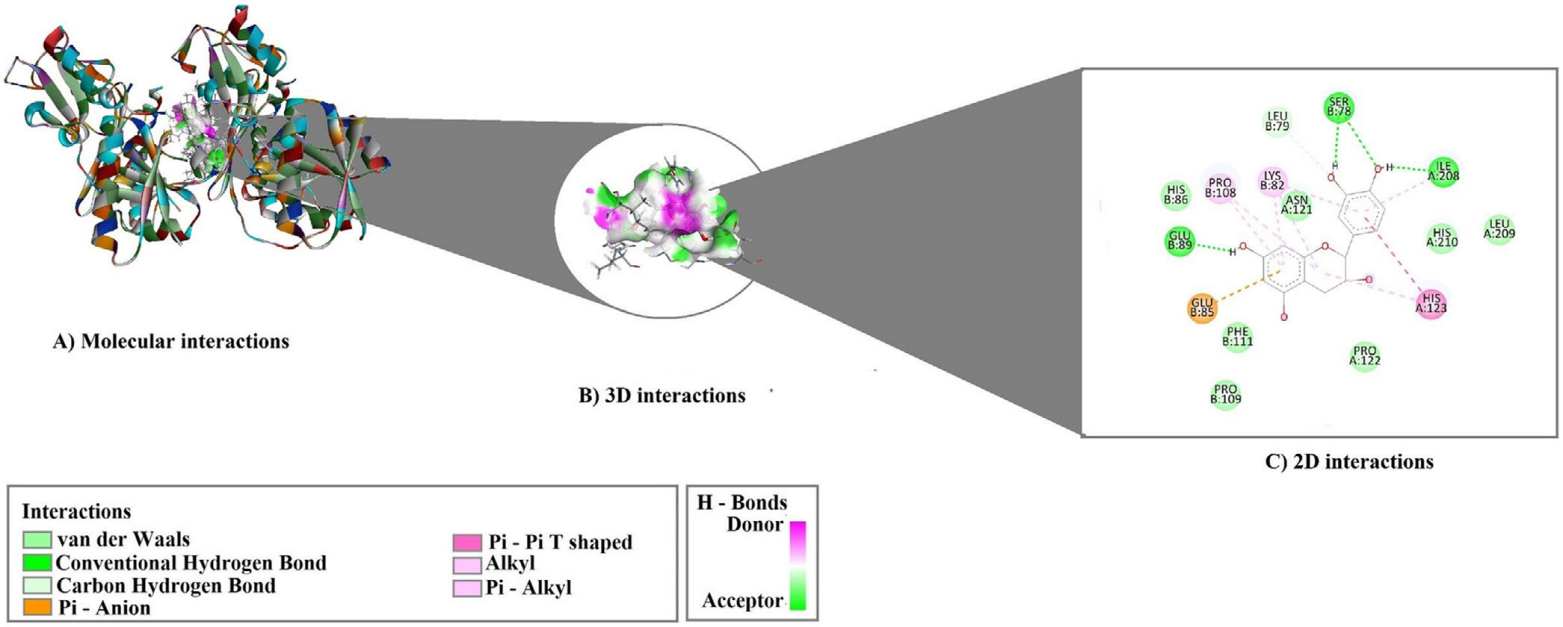
Molecular docking interactions of epicatechin with PBGD (3ECR): (A) molecular interactions, (B) 3D interactions, and (C) 2D interactions.

Molecular docking analysis revealed that both rutin and epicatechin formed multiple non‐covalent interactions with 3ECR (Table 4). The rutin molecule established numerous hydrogen bonds with the target protein, including conventional and C–H type interactions involving residues such as HIS210 HD1–O6, ARG249 HH12–O13, and multiple contacts with GLU89 OE1, GLU85 OE1, SER78, GLU120, ASN121, and PRO122. Additionally, π–π interactions were observed between rutin and TYR62 (two π–π stacked conformations), HIS123, HIS210, and PHE200. A π–anion interaction was also identified with ASP43. In terms of hydrophobic interactions, rutin was involved in alkyl contact with PRO109 and π–alkyl interactions with PRO108, PRO211, ILE208, and LYS82.

**TABLE 4:** Molecular interaction profiles of rutin and epicatechin.

Compounds	H‐Bond	π‐Stacking/π‐electrostatic	Alkyl/π‐alkyl interactions
**Rutin**	HIS210 HD1 – O6 ARG249 HH12 – O13 H1 – GLU89 OE1 H2 – GLU89 OE1 H3 – GLU85 OE1 H5 – HIS271 NE2 H8 – SER78 O H9 – SER78 OG H17 – GLU89 OE2 H18 – GLU89 OE2 ARG119 HD1 – O16 (C–H) ASN121 HA—O2 (C–H) PRO122 HD2 – O12 (C–H) H14 – GLU85 OE1 (C–H) H16 – GLU89 OE1 (C–H) H20 – GLU120 O (C–H) H21 – GLU89 OE2 (C–H)	TYR62 (2 × π–π stacked) HIS123 (π–π T‐shaped) HIS210 (π–π T‐shaped) PHE200 (π–π T‐shaped) ASP43 OD2 (π–anion)	C27 – PRO109 (alkyl) [001] –PRO108 (π–alkyl) [001] –PRO211 (π–alkyl) [001] –ILE208 (π–alkyl) [001] –LYS82 (π–alkyl)
**Epicatechin**	SER78 HG—O6 H2 – GLU89 OE1 H9 – SER78 OG H10 – ILE208 O LEU79 HA—O5 (C–H)	GLU85 OE1 (π–anion) HIS123 (π–π T‐shaped)	LYS82 – [001] (alkyl) PRO108 – [001] (alkyl) HIS123 – [001] (π–alkyl) [001]—LYS82 (π–alkyl × 2) [001]—PRO108 (π–alkyl) [001]—ILE208 (π–alkyl)

Similarly, the epicatechin molecule displayed several key interactions with 3ECR. Conventional and C–H hydrogen bonds were formed with residues such as SER78, GLU89, SER78 OG, ILE208, and LEU79. π‐type interactions included a π–anion contact with GLU85 and a T‐shaped π–π interaction with HIS123. Hydrophobic interactions were also identified, involving alkyl and π–alkyl interactions with LYS82, PRO108, HIS123, and ILE208, with multiple π–alkyl contacts observed particularly with LYS82 and PRO108.

In molecular docking analysis, rutin and epicatechin exhibited binding energies of −9.9 and −7.8 kcal/mol toward PBGD, respectively, indicating favorable predicted binding affinities. These findings suggest that rutin and epicatechin may interact with PBGD; however, the biological significance of these interactions and their possible contribution to light‐enhanced cytotoxicity or photodynamic responses require further experimental validation.

In the present study, rutin, epicatechin, and other phytochemical constituents identified in *E. anacampseros* may have contributed to the observed wavelength‐dependent light‐enhanced cytotoxicity. However, because isolated compounds and ROS‐related mechanisms were not experimentally evaluated, their individual contributions and potential photosensitizing roles remain to be elucidated in future studies. A limitation of the present study is the absence of intracellular ROS measurements and other mechanistic assays. Therefore, although the extract exhibited wavelength‐dependent light‐enhanced cytotoxicity, the precise molecular mechanisms underlying these effects have not yet been experimentally confirmed and warrant further investigation. In addition, the absence of a clinically established PS as a positive control limits direct comparison of the observed light‐enhanced cytotoxicity with current photodynamic agents. Future studies should include reference PSs, such as 5‐ALA, photofrin, or chlorin e6, to facilitate comparative evaluation of the extract's photodynamic potential.

In cancer PDT, the interaction of a PS, light, and oxygen triggers cell death through apoptosis and necrosis. PDT is a treatment modality that exerts selective cytotoxic effects on neoplastic tissues. It has been reported that treatment with 5‐ALA is an effective approach for inducing the accumulation of protoporphyrin IX (PpIX), the final product of the heme biosynthesis pathway, thereby generating tumor‐specific intracellular fluorescence in neoplastic cells [38]. Heme biosynthesis begins with the condensation of two ALA molecules by the cytosolic enzyme ALA dehydratase to form porphobilinogen (PBG). The resulting PBG binds to the active site of PBGD, leading to the activation of the enzyme and the continuation of the porphyrin biosynthetic pathway. In surgical applications, ALA has also been shown to improve the intraoperative visualization of malignant tissues [39]. Increased PpIX accumulation is a common characteristic of tumors in situ. ALA‐mediated photodynamic therapy (ALA‐PDT) enhances PpIX accumulation in neoplastic tissues because of the dysregulated heme biosynthesis pathway, thereby inducing apoptotic and necrotic cell death through photodynamic mechanisms [40].

In the molecular docking analysis, rutin and epicatechin exhibited binding energies of −9.9 and −7.8 kcal/mol, respectively, against PBGD, indicating predicted high binding affinity of both ligands toward the enzyme. Based on these molecular docking findings, rutin and epicatechin present in *E. anacampseros* extract may be considered candidate compounds that could contribute to the potential efficacy of PDT. Nevertheless, it should be emphasized that molecular docking is a computational approach that only predicts potential protein–ligand interactions and does not provide experimental evidence of biological activity.

## 4 Conclusion

In conclusion, the methanolic extract of *E. anacampseros* exhibited wavelength‐dependent light‐enhanced cytotoxicity against HT‐29 cancer cells. Phytochemical characterization identified 22 chemical constituents, while molecular docking analysis indicated that rutin and epicatechin exhibited favorable binding affinities toward PBGD, suggesting that these compounds may be potential contributors to PDT efficacy based on docking predictions. Although the extract alone showed limited cytotoxic activity, light exposure significantly enhanced its cytotoxic effects. While the extract demonstrated potent light‐enhanced cytotoxicity, the low SI between normal and cancer cells highlights the need for targeted application strategies. These findings suggest *that E. anacampseros* represents a potential source of photoactive compounds, though further purification and mechanistic validation are required to improve its therapeutic profile. Furthermore, isolation of the active compounds, biological validation of the docking predictions, and subsequent in vivo studies are warranted to further explore its therapeutic potential.

## Author Contributions

**Erdi Can Aytar**: writing – original draft, software, investigation, formal analysis, supervision, and data curation. **Sercin Ozlem Caliskan**: writing – original draft, visualization, software, investigation, formal analysis, and data curation. **Metin Caliskan**: investigation, software, formal analysis, data curation, and writing – original draft. **Emine İncilay Torunoğlu**: investigation, writing – original draft, visualization, and software. **Abidin Gümrükçüoğlu**: investigation, software, writing – review and editing. **Süleyman Doğu**: investigation, writing – review and editing .

## Funding

The authors have nothing to report.

## Ethics Statement

The authors have nothing to report.

## Consent

The authors have nothing to report.

## Use of Generative AI and AI‐Assisted Technologies in the Writing Process

During the preparation of this work, the author(s) used ChatGPT (OpenAI) to improve the clarity and language of the manuscript.

## Conflicts of Interest

The authors declare no conflicts of interest.

## Supporting information




**Supporting File 1**: cbdv71897‐sup‐0001‐SuppMat.docx.

## Data Availability

The data supporting this study's findings are available from the corresponding author upon reasonable request.
