# Supplementary material for: Photodynamic and Cytotoxic Evaluation of Euphorbia anacampseros Methanolic Extract: Chemical Characterization, Molecular Docking, and Light‐Activated Anticancer Activity
Source: Chem Biodivers. 2026 Oct 30;23(11):e71897. doi: 10.1002/cbdv.71897 (PMC13720957; doi:10.1002/cbdv.71897)
Supplement: Supplementary file 1 [file CBDV-23-e71897-s001.docx]

**Photodynamic and Cytotoxic Evaluation of *Euphorbia anacampseros* Methanolic Extract: Chemical Characterization, Molecular Docking, and Light-Activated Anticancer Activity**

**Sercin Ozlem Caliskan^a^, Metin Caliskan^b^, Emine İncilay Torunoğlu^c^ Abidin Gümrükçüoğlu^d^, Süleyman Doğu^e, f^, Erdi Can Aytar^g^***

^a^Usak University, Faculty of Medicine, Department of Biophysics, Usak, Turkey

^b^Usak University, Faculty of Medicine, Department of Medical Biology, Usak, Turkey

^c^ Necmettin Erbakan University, Faculty of Medicine, Department of Medical Biochemistry, Konya, Türkiye

^d^Artvin Çoruh University, Medicinal-Aromatic Plants Application and Research Center, Artvin, Türkiye

^e^Necmettin Erbakan University, Meram Vocational School Konya, Turkey

^f^Medical and Cosmetic Plants Application and Research Center, Konya, Turkey

^g^Usak University, Faculty of Agriculture, Department of Horticulture, Usak, Turkey

**Address correspondence to:** Dr. Erdi Can Aytar

Usak University, Faculty of Agriculture,

Department of Horticulture, Usak, Turkey

E-mail: [erdicanaytar@gmail.com](mailto:erdicanaytar@gmail.com) , [erdi.aytar@usak.edu.tr](mailto:erdi.aytar@usak.edu.tr)

**Figure S1.** HPLC–DAD chromatograms of the methanolic extract of *Euphorbia anacampseros*: (A) Study A, (B) Study B.

**Figure S2.** HS-SPME–GC–MS total ion chromatogram of the *Euphorbia anacampseros* extract; labelled peaks correspond to Table 3.

**Table S1.** **p**-values were calculated separately for each extract concentration across all groups in HEK293 and HT-29 cells. Only concentrations exhibiting statistically significant differences are presented; concentrations with no significant effect have been excluded for clarity.

| HEK293 | Control | | | | Blue | | | Green | | Yellow |
| --- | --- | --- | --- | --- | --- | --- | --- | --- | --- | --- |
|  | **Blue** | **Green** | **Yellow** | **Red** | **Green** | **Yellow** | **Red** | **Yellow** | **Red** | **Red** |
| 1536 µg/mL | 0.1810 | 0.1207 | **0.0200** | 0.1328 | 0.9985 | 0.6182 | 0.9995 | 0.7697 | 1.0000 | 0.7359 |
| 768 µg/mL | 0.0625 | **0.0275** | **0.0164** | **0.0418** | 0.9822 | 0.9034 | 0.9988 | 0.9968 | 0.9986 | 0.9710 |
| 384 µg/mL | **<0.0001** | **<0.0001** | **<0.0001** | **<0.0001** | 0.7162 | 0.9408 | 0.1774 | 0.9830 | 0.7569 | 0.4687 |
| 192 µg/mL | **<0.0001** | **<0.0001** | **<0.0001** | **<0.0001** | **0.0220** | 0.5411 | **0.0072** | 0.2403 | 0.9428 | 0.0811 |
| 96 µg/mL | **0.0356** | **0.0002** | **0.0042** | **<0.0001** | **0.0226** | 0.6290 | **0.0001** | 0.1977 | **0.0208** | **0.0007** |
| 48 µg/mL | 0.9988 | 0.1496 | 0.4059 | 0.1230 | 0.2173 | 0.5410 | 0.1801 | 0.9429 | 0.9999 | 0.9024 |
| 24 µg/mL | 0.9181 | 0.9303 | 0.9995 | 0.1909 | 1.0000 | 0.9704 | 0.5376 | 0.9767 | 0.5160 | 0.2569 |
| 12 µg/mL | 0.9999 | 0.8824 | 0.9935 | 1.0000 | 0.9317 | 0.9988 | 0.9988 | 0.9835 | 0.8326 | 0.9833 |
| HT-29 | **Control** | | | | **Blue** | | | **Green** | | **Yellow** |
|  | **Blue** | **Green** | **Yellow** | **Red** | **Green** | **Yellow** | **Red** | **Yellow** | **Red** | **Red** |
| 1536 µg/mL | 0.9939 | 1.0000 | 0.9978 | 0.9938 | 0.9897 | 1.0000 | 0.9260 | 0.9957 | 0.9966 | 0.9515 |
| 768 µg/mL | 0.0630 | **0.0081** | **0.0025** | **0.0329** | 0.6747 | 0.2744 | 0.9926 | 0.9235 | 0.8819 | 0.4612 |
| 384 µg/mL | **0.0003** | **<0.0001** | **<0.0001** | **<0.0001** | **0.0115** | **0.0005** | 0.3511 | 0.2169 | 0.2246 | **0.0067** |
| 192 µg/mL | **0.0116** | **0.0002** | **<0.0001** | **0.0017** | 0.0801 | **0.0036** | 0.6679 | 0.3085 | 0.5106 | **0.0270** |
| 96 µg/mL | 0.7465 | 0.0514 | **0.0382** | 0.3263 | 0.3057 | 0.2358 | 0.9239 | 0.9996 | 0.7193 | 0.6115 |
| 48 µg/mL | 1.0000 | 0.8686 | 0.6817 | 1.0000 | 0.8636 | 0.6748 | 0.9999 | 0.9952 | 0.9100 | 0.7420 |
| 24 µg/mL | 1.0000 | 0.2060 | 0.7798 | 0.5481 | 0.2114 | 0.7892 | 0.5584 | 0.7486 | 0.9288 | 0.9925 |
| 12 µg/mL | 0.9998 | 0.9565 | 0.9565 | 0.5700 | 0.9828 | 0.9828 | 0.6577 | 1.0000 | 0.9097 | 0.9097 |
